# HBME1 and CK19 expression in non-invasive follicular thyroid neoplasm with papillary-like nuclear features (NIFTP) vs other follicular patterned thyroid lesions

**DOI:** 10.1186/s12957-021-02258-7

**Published:** 2021-05-08

**Authors:** Qandeel Sadiq, Radhika Sekhri, Daniel T. Dibaba, Qi Zhao, Shweta Agarwal

**Affiliations:** 1 Department of Pathology, Methodist University Hospital, University of Tennessee Health Sciences Center, Memphis, TN USA; 2 Department of Pathology, Montefiore Medical Center/Albert Einstein College of Medicine, New York City, NY USA; 3 Tennessee Clinical and Translational Science Institute, University of Tennessee Health Science Center, Memphis, TN USA; 4 Department of Preventive Medicine, University of Tennessee Health Science Center, Memphis, TN USA; 5 Department of Pathology, University of New Mexico School of Medicine, MSC08 4640, 1 University of New Mexico, Albuquerque, NM 87131 USA

**Keywords:** NIFTP, Papillary, Follicular, HBME1, CK19

## Abstract

**Background:**

Thyroid neoplasms with follicular architecture can have overlapping morphologic features and pose diagnostic confusion among pathologists. Various immunohistochemical stains have been investigated as potential diagnostic markers for PTC, among which HBME1 and CK19 have gained popularity. Non-invasive follicular thyroid neoplasm with papillary-like nuclear features (NIFTP) poses similar diagnostic challenges with interobserver variability and is often misdiagnosed as adenomatoid nodule or follicular adenoma. This study aims to evaluate expression of HBME1 and CK19 in NIFTPs in comparison to other well-differentiated thyroid neoplasms and benign mimickers.

**Method:**

Seventy-three thyroid cases diagnosed over a period of 3 years at Methodist University Hospital, Memphis, TN, USA, were included in this study: 9 NIFTP; 18 papillary thyroid carcinoma (PTC); 11 follicular variant of papillary thyroid carcinoma, invasive (I-FVPTC); 24 follicular adenomas (FA); and 11 multinodular goiters/adenomatoid nodules (MNG). A tissue microarray (TMA) was constructed and HBME1 and CK19 immunohistochemistry was performed.

**Results:**

77.8% of NIFTPs, 88.9% of PTCs, 81.8% of I-FVPTCs, 16.7% of FAs, and 18.2% of MNGs showed HBME-1 expression. 66.7% of NIFTPs, 83.3% of PTCs, 81.8% of I-FVPTCs, 33.3% of FAs, and 45.4% of MNGs expressed CK19. Difference in expression of HBME1 and CK19 was statistically significant for NIFTP vs FA (qualitative; *p* < 0.05) and NIFTP vs MNG (*p* < 0.05). No statistically significant difference was found for HBME1 in NIFTP vs PTC (conventional and FVPTC), *p* ≥ 0.2. Sensitivity of HBME1 and CK19 for NIFTP were 78% and 67%, ~ 88% each for PTC, and 89% and 100% for FVPTC, respectively, while specificity of HBME1 and CK19 for NIFTP were 53% each, ~ 62% each for PTC, and ~55% each for FVPTC.

**Conclusion:**

Our study indicated that HBME1 and CK19 are valuable markers in differentiating NIFTPs from morphologic mimics like follicular adenoma and adenomatoid nodules/multinodular goiter. While HBME1 and CK19 are both sensitive in diagnosing lesions with PTC-like nuclear features, CK19 stains a higher number of benign lesions in comparison to HBME1. No increase in sensitivity or specificity in diagnosis of NIFTP, PTC, or FVPTC was noted on combining the two antibodies.

## Introduction

The rate of thyroid cancer in the USA is escalating rapidly with a reported increase in incidence of 3.6% per annum and the number of newly diagnosed cases going up to 56,430 yearly [1–3]. Papillary thyroid carcinoma (PTC) makes up most of the thyroid malignancies with follicular variant being the most common subtype [4]. The follicular variant of papillary thyroid carcinoma (FVPTC) has become the most common architectural pattern with the percentage rising exponentially from 18 to 57% in the last few decades [5]. FVPTC has two known subtypes — infiltrative and encapsulated, with the latter demonstrating an indolent behavior. The encapsulated form could be invasive or non-invasive [6–12]. Several studies reiterated that non-invasive form of encapsulated FVPTC (NI-EFVPTC) exhibited a behavior comparable to that of benign nodules and was being overtreated [13]. This led to the proposal of the new terminology “Noninvasive follicular thyroid neoplasm with papillary-like nuclear features (NIFTP)” by the Endocrine Pathology Society working group (ESPWG) for these tumors with indolent behavior [13, 14].

Follicular lesions of the thyroid often pose diagnostic dilemmas due to the morphologic resemblance and architectural similarities in benign and malignant lesions. There are studies citing the prevalent interobserver variability in the diagnosis of thyroid lesions. Saxen et al. reported a 58% agreement among pathologists for thyroid tumors [15, 16]. Similar findings have been noted in further studies, especially pertaining to the follicular lesions of thyroid [17–20]. With increasing diagnostic perplexity, the focus shifted to use of immunohistochemical markers to delineate benign from malignant lesions and distinguish the various follicular neoplasms [7, 21–23]. Various immunohistochemical (IHC) stains have been investigated as potential diagnostic markers for PTC, which include CK19, HBME1 (Hector Battifora Mesothelial-1), FN1 (fibronectin1), CITED1 (Cbp/p300-interacting transactivator with Glu/Asp-rich carboxy-terminal domain, 1, also known as melanocyte-specific gene 1), and GAL3 (galectin3) [4, 17]. Among these, HBME1 and CK19 have gained popularity. HBME1 is a monoclonal antibody which is known to act against the microvillous surface of mesothelial cells and has shown to be expressed in thyroid malignancies, while negative in benign lesions [4, 22, 24–26]. CK19 has also proved useful in this regard, exhibiting strong and diffuse expression in thyroid malignancies and focal weak staining in benign nodules [22, 27, 28].

This study aims at investigating expression of these two biomarkers (HBME1 and CK19) in the commonly encountered benign and malignant thyroid nodules in a random and blinded manner. Furthermore, the purpose was to study effectiveness of these markers in differentiating challenging cases of NIFTP from benign entities like follicular adenoma (FA) and adenomatoid nodules. No molecular studies were performed as part of this study.

## Material and methods

### Case selection

After obtaining approval of this retrospective study by International Review Board (IRB) of University of Tennessee Health Sciences Center (UTHSC), the Methodist University hospital database was queried for thyroid cases belonging to the following categories: multinodular goiter (MNG)/adenomatoid nodules, FA, papillary thyroid cancer (PTC), invasive form of follicular variant of papillary thyroid carcinoma (I-FVPTC), and non-invasive follicular thyroid neoplasm with papillary-like nuclear features (NIFTP).

Benign thyroid nodules with variably dilated follicles, absent thick capsule, and flattened to hyperplastic lining epithelium were included under the MNG/adenomatoid nodule category. Solitary encapsulated thyroid nodules, architecturally and cytologically different from surrounding gland and lacking nuclear features of PTC, were included as FAs in this study. Malignant thyroid cases with classical nuclear features of PTC (nuclear enlargement, elongation and overlapping, chromatin clearing, nuclear membrane irregularity, nuclear grooves, and nuclear pseudoinclusions) showing complex papillary architecture were categorized as PTC. Variants like hobnail, tall cell and diffuse sclerosing were also included in the PTC category.

Since this study aims to analyze the diagnostic performance of HBME-1 and CK19 in distinguishing follicular patterned lesions, we categorized FVPTC variant as a separate category. Cases of PTC with follicular growth pattern showing infiltration into the surrounding thyroid were included as FVPTC-I while NIFTP/encapsulated FVPTC cases were selected based on the revised, universally accepted, specific inclusion/exclusion criteria published in 2019 [6].

A total of 73 cases over a period of 3 years from 2016 to 2019 were identified. Clinical data including age, gender, and size of tumor was recorded for each case. Detailed review of H&E slides was conducted by a Head and Neck Pathologist (SA) to characterize the tumors in each category. A change in the primary diagnosis in three cases was rendered as follows: Two cases of FVPTC were reclassified on review: (1) as NIFTP, due to presence of a capsule and absence of invasion and (2) as PTC, due to presence of mixed papillary and follicular architecture, while one case previously diagnosed as NIFTP was altered to I-FVPTC (due to presence of infiltration), making the total number as follows: MNG (*n* = 11) including hyperplastic and adenomatoid nodules, FA (*n* = 24) [4 Hürthle cell/oncocytic type, 1 of clear cell and macro-follicular type each, and 18 microfollicular], PTC (*n* = 18) [usual = 13, hobnail = 2, focal tall cell features = 2, diffuse sclerosing = 1], FVPTC (*n* = 11), and NIFTP (*n* = 9).

### Tissue microarray (TMA)

H&E slides were reviewed by the pathologist to select the best possible area representative of the diagnosis for the TMA. Formalin-fixed paraffin-embedded tissue blocks were then utilized to construct 1-mm single cores (*n* = 73) using a semi-automated tissue microarrayer (Advanced Tissue Arrayer from Veridiam) to evaluate the immunohistochemical expression.

### Immunohistochemistry (IHC)

IHC for HBME1 (Cell-Marque pre-dilute Clone HBME1) and CK-19 (Roche Pre-dilute Dispenser Clone = A53-B/A2.26) was performed on the TMA created from paraffin-embedded tissue blocks of 73 selected cases at PathGroup, TN. IHC staining of all the cores was then analyzed in a random and blinded fashion. HBME1 and CK19 were considered positive if more than 10% lesional cells showed membranous and membranous/cytoplasmic staining, respectively.

### Statistical analysis

Positive/negative IHC results (HBME1/CK19) were taken as categorical variables and analyzed by Chi-square analysis; result was expressed as a percentage (qualitative). Categorical variables were summarized as count and percentage and compared between disease types using the *χ*^2^ test. The association between HBME1 and CK19 expression and disease as a binary outcome (NIFTP, PTC, FA, etc.) was conducted using the logistic regression analysis adjusting for demographic variables.

The following analyses were performed:

1. Expression of HBME1 and CK19 in NIFTP was compared to that observed in other well-differentiated thyroid neoplasms and *p* value was calculated.
2. HBME1 and CK19 were cross classified, and their expression (cumulative) was compared between two diagnoses (NIFTP vs PTC, NIFTP vs FA, NIFTP vs MNG, and NIFTP vs FVPTC) and *p* value was calculated.
3. HBME1 and CK19 were cross classified, and their expression (cumulative) was compared between all diagnoses and *p* value was calculated.

A *p* value ≤ 0.05 is interpreted as statistically significant.

A *p* value > 0.05 is interpreted as not statistically significant.

In addition, sensitivity, specificity, and predictive values for HBME1 and CK19 in diagnosing NIFTP were calculated. The association between HBME1 and CK19 expression and disease types as a binary outcome (NIFTP, PTC, FA, etc.) was conducted using the logistic regression analysis adjusting for demographic variables. The receiver operating characteristics (ROC) curves were produced for the prediction of the outcomes by the HBME1 and CK19 adjusting for covariates. All the statistical analysis was conducted using R version 3.5.3 (11 March 2019).

## Results

### Demographic data and immunohistochemical expression

Table 1 summarizes the demographic data along with tumor size for the cases included in this study (*n* = 73). Results for HBME1 and CK19 immunohistochemical expression (number and percentage of positive cases) in the different diagnostic categories are summarized in Table 2.

**Table 1:** Demographic data and tumor size of thyroid neoplasms included in the study

	NIFTP (***n*** = 9)	PTC (***n*** = 18)	FVPTC (***n*** = 11)	FA (***n*** = 24)	MNG (***n*** = 11)
F/M	3.5:1	2:1	4.5:1	3:1	4.5:1
Age range (years)	41–72	23–80	21–84	33–72	25–80
Tumor size (range in cm)	0.2–6.5	0.1–7.0^a^	0.3–4.5^a^	0.6–6.2	2.7–7.6^a^

^a^Largest tumor nodule was considered in multifocal cases

**Table 2:** Immunohistochemical expression (qualitative) of HBME1 and CK19 in various well-differentiated lesions

Diagnostic category	Number of cases (***n***)	HBME1+ (***X***)	HBME1+ (%)	CK19+ (***Y***)	CK19+ (%)
NIFTP	9	7	77.8	6	66.7
PTC	18	16	88.9	15	83.3
FVPTC	11	9	81.8	9	81.8
FA	24	4	16.7	8	33.3
MNG	11	2	18.2	5	45.4
**Total**	73	38	N/A	43	N/A

*X* number of cases showing HBME1 positivity, *Y* number of cases showing CK-19 positivity

### Statistical analysis

#### Comparing NIFTP with each separate diagnoses

1. *FA*. 16.7% expressed HBME1, while 33.3% showed positive expression for CK19 vs 77.8% and 66.7% for NIFTPs, respectively. The difference was found to be statistically significant for both antibodies (*p = 0.002 for HBME1 and p = 0.02 for CK19*)*.*
2. *MNG*. 18.2% expressed HBME1 while 45.4% showed positive staining for CK19. The difference was statistically significant in both percentage (*p* = 0.008, HBME1; *p* = 0.009, CK19).
3. *PTC*. No statistically significant difference was found for either HBME1 or CK19 expression between PTC and NIFTP.
4. *FVPTC*. No statistically significant difference was observed.

**Fig. 1:**
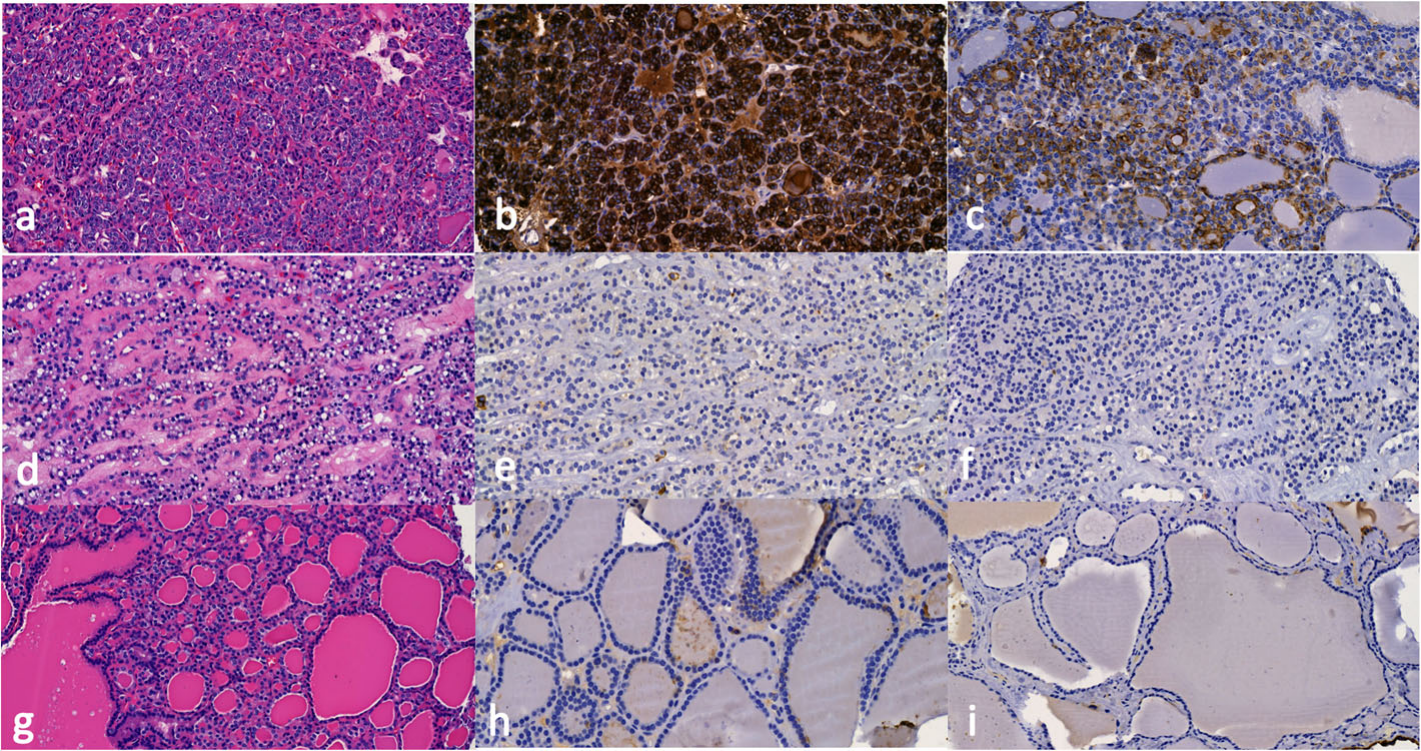
Representative histologic images of NIFTP, FA, and MNG (**a**, **d**, **g**). **b**, **e**, **h** The differential expression of HBME1. **c**, **f**, **i** Expression of CK19 in NIFTP, FA, and MNG, respectively. Magnifications in **a**, **d**, **f**, **g**, and **i** are × 22 and **c** and **e** are × 25 while **b** and **h** are × 30

**Fig. 2:**
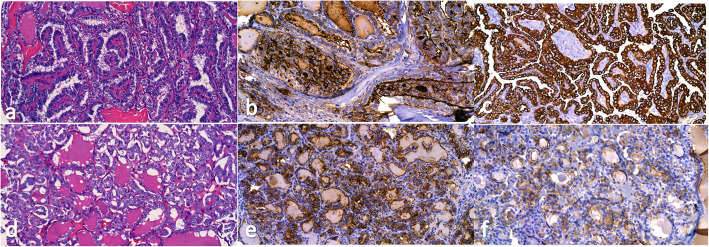
Representative histologic images of PTC-c and FVPTC (**a**, **d**). **b**, **e** The differential expression of HBME1. **c**, **f** Expression of CK19 in PTC and FVPTC, respectively. Magnifications in **a**, **c**, and **d** are × 22 and **f** is × 25 while **b** and **e** are × 30

Figure 1 shows the histologic features and immunohistochemical expression of HBME-1 and CK19 in NIFTP vs FA and MNG. Figure 2 shows histologic features and the immunohistochemical expression of HBME-1 and CK19 in PTC and FVPTC.

#### Cross classification of HBME1 and CK19 and two-way diagnosis

This method of data analysis showed statistically significant results for NIFTP vs FA (*p* = 0.002) and NIFTP vs MNG (*p* = 0.005), while no significance was found for NIFTP vs PTC and/or NIFTP vs FVPTC.

#### Cross classification of HBME1 and CK19 and all diagnosis

**Fig. 3:**
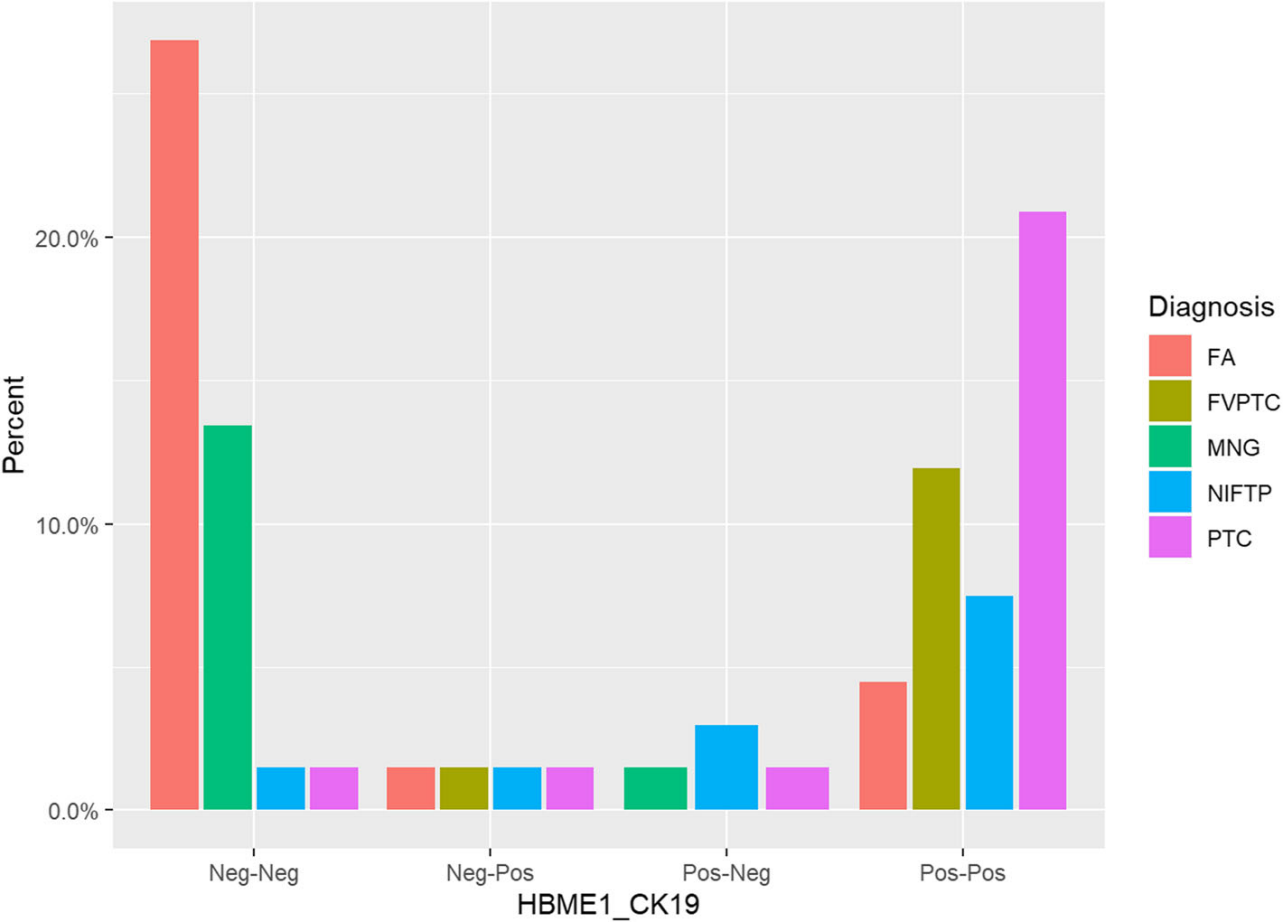
Bar diagram showing all diagnosis and cross classification of HBME1 and CK19

This method showed a *p* value of < 0.0001, which indicated highly significant results. Figure 3 shows the distribution of all diagnoses with cross classification of HBME1 and CK19. It is quite evident from the bar diagram that benign diagnoses like FA and MNG are clustered on the left-hand side of the graph with most cases staining negative for both antibodies (HBME1-CK19: Neg-Neg) while PTC and NIFTP have a higher distribution along the right-hand side of the graph (HBME1-CK19: Pos-Pos).

#### ROC curve analysis results

Tables 3, 4, and 5 summarize the sensitivity, specificity, positive predictive value, and negative predictive value of HBME1 and CK19 (as calculated by the ROC curve analysis and DeLong’s test) for NIFTP, PTC (classical), and I-FVPTC, respectively.

**Table 3:** Sensitivity, specificity, and predictive values of HBME1 and CK19 in diagnosis of NIFTP

	Sensitivity	Specificity	Positive predictive value	Negative predictive value
**HBME1**	0.78	0.53	0.19	0.94
**CK19**	0.67	0.53	0.18	0.91
**HBME1 + CK19**	0.56	0.57	0.17	0.89

**Table 4:** Sensitivity, specificity, and predictive values of HBME1 and CK19 in diagnosis of PTC

	Sensitivity	Specificity	Positive predictive value	Negative predictive value
**HBME1**	0.89	0.62	0.44	0.94
**CK19**	0.88	0.63	0.44	0.94
**HBME1 + CK19**	0.82	0.68	0.47	0.92

**Table 5:** Sensitivity, specificity, and predictive values of HBME1 and CK19 in diagnosis of FVPTC

	Sensitivity	Specificity	Positive predictive value	Negative predictive value
**HBME1**	0.89	0.55	0.24	0.97
**CK19**	1.0	0.57	0.26	1.0
**HBME1 + CK19**	0.89	0.62	0.27	0.97

## Discussion

Papillary thyroid carcinoma is usually a morphologic diagnosis with characteristic nuclear features such as large, overlapping, ground glass nuclei, nuclear grooves, and pseudo inclusions and rarely requires immunohistochemistry to confirm the diagnosis. Histologically, classic PTC and follicular variant are the two major low-risk subtypes of PTC with other high-risk variants like tall cell, diffuse sclerosing, and hobnail variants reported in literature [29, 30].

FVPTC encompasses a wide spectrum of morphology ranging from micro- to macro-follicular and diffuse growth pattern and could be encapsulated or infiltrative often creating diagnostic confusion with other follicular neoplasms. Tallini et al. [31] in 2017 published a detailed historical review of the emergence of the term “Follicular Variant of Papillary Thyroid Carcinoma”. FVPTC was first officially defined by Chen and Rosai [32] in 1977 after Lindsay found papillary carcinoma-like nuclear features in a subset of follicular carcinomas [29]. In 1980s, the encapsulated variant of FVPTC was recognized. This led to the classification of thyroid tumors showing predominant follicular growth pattern with nuclear characteristics of PTC into 3 main groups: (1) encapsulated FVPTC without invasion (EFVPTC), and (2) encapsulated FVPTC with capsular and/or vascular invasion and infiltrative FVPTC without a tumor capsule [31]. In 2016, non-invasive EFVPTC was re-categorized as non-invasive follicular thyroid neoplasm with papillary-like nuclear features (NIFTP) by Nikiforov et al. [13].

Since the new classification in 2016, several studies have evaluated biologic behavior of NIFTPs. Analysis of 94 NIFTP cases by Thompson et al. [33] and 129 cases by Rosario et al. [7] supported the low-risk behavior and conservative approach in treating the patients with NIFTP. Molecular studies on the encapsulated/well-circumscribed FVPTCs have found primarily RAS mutations and thereby suggested their close relationship with other follicular neoplasms of the thyroid such as follicular adenoma and follicular carcinoma [34–37].

Follicular patterned lesions of the thyroid have high level of interobserver as well as intraobserver disagreement [37, 38]. A considerable degree of discordance has been reported among pathologists in the diagnosis of FVPTC, the encapsulated type, in particular [20, 38]. The diagnostic criteria for NIFTP includes encapsulated/well-demarcated tumor without any invasion, no papillary growth, no evidence of psammomatous calcifications or tumor necrosis, < 30% solid/trabecular or insular growth pattern, and nuclear features of PTC with nuclear score of 2–3 [13, 34].

A high degree of interobserver variability has been observed, even among expert pathologists as the nuclear features of PTC could be only focal/subtle [13, 38]. Unfortunately, there are no established criteria like required percentage of the follicular neoplasm showing nuclear features of PTC and/or the more reliable nuclear features (overlapping vs irregular nuclear outlines) that can help diagnose this entity as EFVPTC vs FA [38, 39]. NIFTP is still an evolving diagnosis and the struggle in diagnosing this entity is real.

Immunohistochemistry, although seldom required, can be helpful in differentiating FVPTC from other follicular lesions [26, 40, 41]. Various IHC markers have been explored to characterize the immunohistochemical profile of thyroid tumors especially the follicular patterned lesion which causes significant diagnostic confusion with high rate of interobserver disagreement. Among these, most notable are HBME1, Cytokeratin 19 (CK19), galectin-3 (GAL3), CITED1, and Thyroid peroxidase (TPO). HBME1 (Hector Battifora Mesothelial-1), a monoclonal antibody directed against microvilli and a marker of mesothelial and other epithelial cells, has shown significant expression in malignant thyroid with a sensitivity of 78.8% for thyroid malignancy, 87.3% for PTC, and 65.2% for follicular carcinomas and specificity of 82.1% [37, 42]. In our study, sensitivity and specificity of HBME-1 for PTC was found to be 89% and 62%, respectively, and 89% and 55% for FVPTC, respectively.

CK19 is a low molecular weight cytokeratin which is demonstrated in both simple as well as complex epithelium and has been widely utilized in thyroid neoplasms [17, 21, 43]. Baloch et al. [44] employed a panel of cytokeratins including CK5/6 and CK 18, 10/13, 14, 17, 18, 19, and 20 in FVPTC. The authors found that CK19 was useful in diagnosis of PTC (showed diffuse staining pattern), only focally expressed in follicular tumors, but was expressed in normal thyroid tissue. It has also been proven a helpful marker in cytology specimens of unequivocal cases of PTC [28, 45]. Khurana et al. [45] reported a sensitivity and specificity of 93% and 100% which was comparable to that reported by Nasser et al. [28]. In our series, the sensitivity and specificity of CK19 in diagnosis of PTC were 88% and 63%, respectively, while the values were 100% and 57% for diagnosis of FVPTC. Although the staining was weak to moderate in intensity, we did see about 33% FAs and 45% MNG nodules showing CK19 expression. Our findings agree with those of Baloch et al. and Haiyan Lu et al. [37, 40] who also found CK19 expression in normal thyroid parenchyma. We did not find any difference in antigen localization among positive malignant vs positive benign cases. Similar to our findings, Casey et al. [46] also reported weak to moderate positive expression of CK19 in 12/30 benign thyroid cases with papillary hyperplasia with a sensitivity and specificity of 100% and 60% for PTCs.

In our study, we found a significant difference in the expression of CK19 and HBME1 in NIFTP cases in comparison to other benign follicular lesions (*p* < 0.02 for both markers). HBME1 was expressed in 77.8% cases of NIFTP, while only 16.7% and 18.2% cases of FA and MNG showed positive staining, respectively. Frequent expression was also noted in cPTC (88.9%) and FVPTC (81.8%) cases which agree with the percentage reported in literature [37, 47]. Gucer et al. [48] reported an expression score of 77% for HBME1 in non-invasive RAS like PTCS/NIFTPs, corroborating with our findings (77.8% for NIFTPs in our study). Further, the sensitivity of HBME1 and CK19 in our study was found to be 78% and 67%, respectively, for diagnosis of NIFTP, while specificity was 53% for both biomarkers. To the best of our knowledge, this is the first study evaluating diagnostic value of HBME-1 and CK19 in NIFTP in comparison to other benign and malignant follicular patterned neoplasms of the thyroid gland.

Similarly, CK19 showed higher expression in NIFTP (66.7%), cPTC (83.3%), and FVPTC (81.8%) in comparison to FA (33.3%) and MNG (45.4%). Similar findings have been reported by Liu et al. [49] who reported statistically significant expression of CK19 and HBME1 in PTC vs benign thyroid lesions. Sensitivity of CK19 and HBME1 in diagnosis of PTC was reported to be 96.30% and 85.3%, respectively, while the reported specificity was 40% and 62%, respectively [49]. Our series reported a sensitivity and specificity of ~ 89% and ~ 63%, respectively, for both antibodies in diagnosis of PTC (Table 4). For FVPTC, the sensitivity of HBME1 and CK19 was found to be 89% and 100%, respectively, while specificity was ~ 55% for both antibodies (Table 5).

HBME1 was found to be the most sensitive marker of thyroid malignancy by Palo et al. [50], followed by CK19, in differentiating FVPTC from FA and follicular carcinoma. Palo et al. reported an increase in sensitivity with combined use of HBME1 and CK19 in differentiating benign from malignant thyroid lesions [94% with combined use vs 86% (HBME1) and 75% (CK19)]. Saleh et al. [47] did not report increase in sensitivity or specificity with combined use of CK19 and HBME1 vs isolated use of either biomarkers. Findings in our series were concordant with Saleh et al.’s findings and showed no increase in the sensitivity when combining the two antibodies.

Liu et al. [37] published a review article in 2015 in which they analyzed various studies evaluating role of IHC in diagnosing thyroid lesions. The authors concluded that there is no single biomarker sufficient to differentiate between benign and malignant thyroid lesions. Their review found strong and diffuse HBME1 expression, while CK19 had low sensitivity as well as specificity for papillary thyroid carcinomas. The authors suggested including TROP2 (trophoblastic cell surface antigen 2) in the panel along with HBME1, CK19, and Galectin-3 as an aid in diagnosis of PTCs. Our study showed almost similar sensitivity and specificity for HBME-1 and CK19 in diagnosis of PTC and FVPTC as well as NIFTP cases with no added benefit of combining the two antibodies. However, in agreement with Liu et al.’s findings, we found HBME-1 to be a better marker of PTC/FVPTC/NIFTP than CK19, due to the latter showing positive expression in a significant percentage of benign cases (33% FAs and 45% MNG/adenomatoid nodules in this study).

Our study has some limitations, and the findings need further validation. First, our sample size is small with limited number of NIFTP cases (*n* = 9). Second, we recognize that the study used TMA for IHC analysis, and the results might not be completely generalizable as some of these lesions can exhibit heterogeneity for antigen expression.

## Conclusion

Thyroid lesions with follicular architecture have several overlapping histologic features with problems arising particularly in differentiating encapsulated FVPTC/NIFTP from follicular adenomas or adenomatoid nodules in MNG. Our study revealed that HBME1 and CK19 are sensitive markers for diagnosis of NIFTPs, PTC, and FVPTC and can help in rendering the correct diagnosis in challenging cases of EFVPTC without invasion and/or NIFTP with focally developed PTC-like nuclear features. Further, our statistical analysis did not find added significance of combining these two markers in aiding the diagnosis of NIFTP/PTC or FVPTC. We acknowledge that the sample size of this study is small and further studies with larger number of cases (particularly NIFTP) are needed to further validate the findings. Nevertheless, the entire tumor capsule interface should be examined to rule out capsular and/or vascular invasion to avoid missing diagnosis of invasive carcinoma.

## Data Availability

All data generated or analyzed during this study are included in this published article.

## Abbreviations

SA: Shweta Agarwal
QS: Qandeel Sadiq
RS: Radhika Sekhri
DTD: Daniel T Dibaba
QZ: Qi Zhao
NIFTP: Non-invasive follicular thyroid neoplasm with papillary-like nuclear features
TMA: Tissue microarray
MNG: Multinodular goiter
FA: Follicular adenoma
PTC: Papillary thyroid carcinoma
FVPTC: Follicular variant of papillary thyroid carcinoma
IHC: Immunohistochemistry
Pos: Positive
Neg: Negative
